# Early COPD Diagnosis in Family Medicine Practice: How to Implement Spirometry?

**DOI:** 10.1155/2014/962901

**Published:** 2014-04-03

**Authors:** Nathalie Saad, Maria Sedeno, Katrina Metz, Jean Bourbeau

**Affiliations:** Respiratory Epidemiology and Clinical Research Unit (RECRU), Montreal Chest Institute, McGill University Health Centre, Montréal, QC, Canada H2X 2P4

## Abstract

*Introduction*. COPD is often diagnosed at an advanced stage because symptoms go unrecognized. Furthermore, spirometry is often not done. * Methods*. Study was conducted in diverse family medicine practice settings. Patients were targeted if respiratory symptoms were present. Patients had a spirometry to confirm the presence of airflow obstruction and COPD diagnosis. An evaluation of the process was done to better understand facilitating/limiting factors to the implementation of a primary care based spirometry program. * Results*. 12 of 19 primary care offices participated. 196 of 246 (80%) patients targeted based on the presence of smoking and respiratory symptoms did not have COPD; 18 (7%) and 32 (13%) had COPD, respectively, GOLD I and ≥II. There was no difference in the type and number of respiratory symptoms between non-COPD and COPD patients. Most of the clinics did not have access to a trained healthcare professional to accomplish spirometry. They agreed that giving access to a trained healthcare professional was the easiest and most reliable way of doing spirometry. * Conclusion*. Spirometry, a simple test, is recommended in guidelines to make the diagnosis of COPD. The lack of allocated time and training of healthcare professionals makes its implementation challenging in family medicine practices.

## 1. Introduction

Chronic obstructive pulmonary disease (COPD) is a major health problem. COPD will be the fifth leading cause of disability and the third leading cause of death in the coming decades [1]. Treatments are known to be beneficial to COPD patients by improving symptoms and health status, reducing exacerbations and hospital admissions [2, 3]. Although the evidence does not support treating asymptomatic patients [2], symptomatic patients with undiagnosed COPD should respond to treatment like those who have been diagnosed.

The irony is that COPD does not get much attention from healthcare professionals. It is still underdiagnosed in family medicine practices [4] and a major contributing factor has been the underutilization of spirometry [5, 6]. The diagnosis of COPD is often made when the disease is advanced [7, 8]. This could be due to the fact that patients are not complaining early on as they adapt to the gradual decrease of their lung functions [8, 9]. It may also be due to the physicians not being aware of the symptoms or risk factors of their patients [7, 9]. Suspecting COPD in patients with current or past exposure to cigarettes and those with respiratory symptoms is essential.

Spirometry is most important to confirm the presence of airflow obstruction and to assess severity. Despite spirometry being strongly recommended in patients with respiratory symptoms by the ACP, ACCP, ATS, ERS, and GOLD, many physicians rely on clinical examination only. Prediction of airflow obstruction based on clinical examination [10] and pulmonary auscultation [11] can be inaccurate and could lead to inappropriate treatment. Even in developed countries, the number of spirometry tests performed in primary care only increased marginally [12, 13]. It is evident that spirometry is not routinely used to diagnose COPD in primary care [14, 15].

We carried out a fieldwork evaluative project in different family medicine practices to assess the implementation of spirometry testing combined with a simple clinical assessment tool for the early diagnosis of COPD “target testing” in patients not known and not already treated for a chronic respiratory disease. We also wanted to evaluate whether patients' symptoms at presentation could be used to discriminate patients with or without COPD.

## 2. Methods

This is a multisite cross-sectional study conducted in family medicine practices of the territory of the health agency of Montreal between July 2004 and February 2008. The study was approved by the respective Ethics Committee at each of the clinics and at the McGill University Health Centre (MUHC).

### 2.1. Family Medicine Practices and Patients

A family medicine practice was defined as a group of 3 or more family physicians working together. The present study recruited patients from 3 different family practice settings: (1) university hospital family medicine clinic; (2) local community clinic “centre local de services communautaires (CLSC)”; (3) private clinic of family physicians in group practice. The university family medicine clinics were chosen because of the assumption that in this environment diagnostic tests are readily accessible, guidelines are known, and hence spirometry could be part of regular practice. CLSCs were chosen because they offer outpatient services and include a team of healthcare professionals (e.g., nurses), who are already involved in the screening and care of patients with chronic diseases. Private clinics also have the support of a nurse and they are usually taking care of the largest volume of patients.

All patients were assessed by their family physicians and were excluded if they had a previous diagnosis of COPD and/or were on respiratory medications. A short algorithm was developed for the study to determine if patients were eligible for spirometry testing: the COPD Early Diagnosis (CED) tool. Patients fulfilling these criteria were asked to perform a spirometry: (a) age ≥ 40 years, (b) smoking history of ≥10 pack-years, and (c) at least one respiratory symptom (daily cough with or without sputum production, dyspnea on exertion, wheezing, or frequent respiratory infections).

### 2.2. COPD Diagnosis

#### 2.2.1. CED (COPD Early Diagnosis)

Based on the CTS recommendations [3], we implemented a simple tool with standardized questions, the CED, to allow the identification of the clientele at risk of COPD. The elements included in the CED tool were (1) age ≥ 40 years; (2) smoking history, that is, smokers and ex-smokers ≥ 10 pack-years (population at the highest risk); and (3) at least one of the following symptoms: daily cough with or without sputum production, dyspnea on exertion (2 or more on the MRC scale [16]), wheezing, or frequent respiratory infections during the year. If the patient obtained a positive mark for these three criteria he/she was asked to undergo a spirometry test. To create awareness about the importance of spirometry testing in the participating clinics, posters with the CED questions were placed in the waiting rooms and physician offices. Patients who thought they fulfilled the criteria as seen on the poster were encouraged to approach their physician to discuss the need for a spirometry test.

#### 2.2.2. Spirometry

All family practices received spirometers for the project duration if not already in the clinic. A 6-hour training was provided to the healthcare professionals involved in the project on how to perform spirometry, quality control, and study process. A visual instruction guide was developed and provided to healthcare professionals. Although the physicians were not required to interpret the spirometry, a 2-hour training session on interpretation was offered to them. For each patient who tested positive on the CED tool, a postbronchodilator spirometry was performed. Results of the spirometry were then reviewed by a respirologist and the COPD diagnosis was made based on the respirologist diagnosis.

### 2.3. COPD Classification

Patients were classified using the GOLD 2007 criteria [17]. “No COPD” was defined as patients who had a ratio postbronchodilator FEV_1_/FVC ≥ 0.7. “COPD” was defined as patients having a ratio postbronchodilator FEV_1_/FVC < 0.7 and FEV_1_ ≥ 80% of predicted normal value, that is, GOLD stage I, and FEV_1_ < 80% of predicted normal value, that is, GOLD stage ≥II [18].

### 2.4. Program Evaluation and Analysis

The evaluation of the implementation of the program at each of the participating family practices for the COPD clientele, that is, CED tool and spirometry testing, included the participation rate of the clinics and reasons for not participating, the review of the participating clinics and their context of practice, and the facilitating and limiting factors. For the analysis of symptoms' presentation and their discriminative value, frequency of symptoms was assessed based on the absence of COPD (No COPD) and the presence of COPD GOLD I and ≥II. Symptoms at presentation and cumulative number of symptoms were analyzed to assess statistical relevance. Chi-square test (fisher's exact test results reported only frequencies less than 5), *t*-test (normal distributions), and Wilcoxon two-sample test (abnormal distributions) were performed to calculate the *P* values. SAS version 9.2 was used to do the analysis.

## 3. Results

### 3.1. Recruitment of Family Practices and Patients

**Figure 1:**
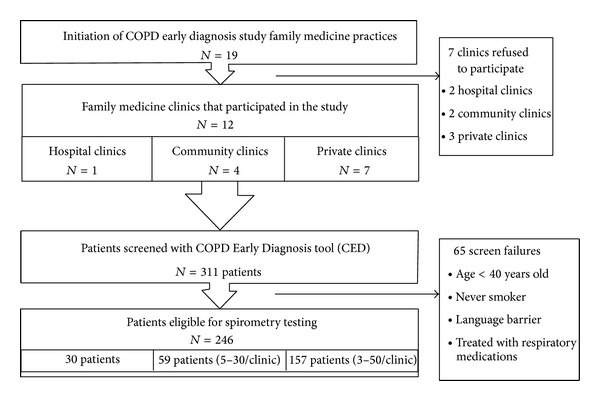
Distribution of the recruitment centers and their patient enrolment.

Out of 19 family medicine practices invited to participate, 12 (63%) agreed to take part in the study (Figure 1), comprising 1 hospital clinic, 4 local community clinics (CLSC), and 7 private clinics. The main reasons for declining participation were no direct access to a pulmonary function laboratory, problems of organization, and lack of interest. Across the 12 participating clinics, a total of 90 general practitioners agreed to take part in the study. Three hundred eleven patients were assessed with the CED, 65 were screen failure, and 246 were eligible for spirometry.

### 3.2. Clinic Settings and Implementation Process

There was a large variation in the number of physicians per practice and in the organization structure across the different environments. Most clinics had access to at least one nurse. Spirometry was done by the nurse in only 2 of the community clinics and none of the hospital or private clinics. Even if nurses had received the training provided by the study, it became evident that they were overloaded with other tasks having to follow up a large clientele (e.g., blood tests, patient education, etc.). Furthermore, because the nurses did not perform the spirometry test often enough, they did not feel comfortable with it. As part of the solutions implemented early in the process, we provided to each of the clinics the services of a respiratory therapist (0.5–1 day/week, based on needs). All the practices agreed that this was the easiest way of doing the spirometry. The limiting and facilitating factors to spirometry implementation are presented in Table 1 according to each family practice setting.

### 3.3. Diagnosis of COPD and Patient Characteristics

One hundred ninety-six (196) of the 246 patients had “No COPD” (80%), while 18 and 32 had COPD GOLD I (7%) and GOLD ≥II (13%). Patients from the “No COPD” group were younger, had less exposure to cigarette, and hence had a higher postbronchodilator FEV_1_/FVC ratio and FEV_1_ (Table 2).

Table 2 also shows the respiratory symptoms at presentation for patients from the “No COPD” group and for those from the “COPD”. The type and number of respiratory symptoms reported were not different between the “No COPD” and the “COPD” patients.

## 4. Discussion

This study showed that implementing a program with identification of patients at risk and target testing with spirometry was challenging but feasible in family medicine practices. The most effective way of achieving spirometry was having a respiratory therapist in the clinic to off load the physicians and the nurses. It was also demonstrated that symptom presentation could not discriminate patients with COPD from those without COPD.

Our study is one of the first to assess and report on the implementation of spirometry in family medicine practice according to various settings of practice. Previous studies have looked at the use of spirometry in family medicine clinics from different angles. Some have analyzed the role played by the treating team and required a nurse to perform the test [19] while others have not considered prescreening the patients to identify the ones at risk for COPD and did spirometry on all participants [5, 20]. We took a different approach, acknowledging the fact that any added test performed during a clinic is time consuming and that the test should be used towards a targeted population at high risk of having COPD. Targeting patients with a questionnaire based on the Canadian Lung Health test and then performing spirometry likely avoided unnecessary prescriptions of respiratory medication. Our study did not intend to assess the appropriateness of treatment of COPD. This is known as a potential problem and studies have shown a rate as high as 40% of inappropriate prescription of inhaled medications in family medicine practice [5, 20, 21].

A gap has been identified in the literature about the need to assess whether or not the symptoms could be used in primary care setting to diagnose COPD [22]. There is still a belief by certain family physicians that spirometry may not be required for patients with a smoking history and presenting with respiratory symptom. Our study reinforces the need of spirometry to establish a COPD diagnosis, as symptoms alone are not enough. A tool like the CED could be used to standardize the need for a diagnostic spirometry to be performed. The most recent GOLD recommendations for the diagnosis and management of COPD have stated that spirometry is required to make a clinical diagnosis of COPD (http://www.goldcopd.org/uploads/users/files/GOLD_Report_2011_Feb21.pdf).

An important strength of this study is its generalizability in real practice. Contrary to many previous studies that looked at secondary care settings or at spirometry done outside of a regular appointment, we focused on implementing a system within real life primary care practices. This study highlights the fact that implementing spirometry should not be considered as an easy process in primary care even in those practices that have the support of health professionals. These professionals not only need to be trained but also require allocated time and may not be confident enough to maintain that role if they do not have sufficient exposure. Other challenges inherent to spirometry are the calibration that is not routinely done and can lead to false measurements [23] and short expiration time that is insufficient to produce valid tests [24]. These obstacles slowed down implantation of spirometry testing in the primary care setting in many countries. Most of the family practices in our study agreed that it was best to have someone trained and dedicated to performing spirometry. This facilitated the accessibility to the test and hence should be encouraged.

When looking at the participation rate, 7/19 (37%) of the clinics approached refused to participate. Hospital based clinics had access to spirometry and respirologists readily and hence considered that spirometry in the clinic was not an added benefit. Therefore spirometry testing programs should be individualized according to the existing hospital setting. For the community and the private clinics the number of patients was an issue. In thinking about implementing a spirometry testing program, this study stresses the role of prioritizing the clinic. The service of a trained and designated healthcare professional should be targeted to the high volume clinics. For the low volume clinic, the minimal exposure and the small number of patients may limit the possibility of having a healthcare professional that provides the service of spirometry within the family clinic.

Although some important conclusions can be drawn from this study, it is worth appreciating the limitations. The spirometry was not interpreted by the family physician and, upon implementing a program similar to the one described, further work should be done to analyze the best way of having the spirometry results interpreted. Some studies have looked at the interpretation of spirometry results by family physicians and found high misdiagnosis rate [20, 21] and our study did not address this question. Another limitation is that we did not carry out interviews to assess qualitatively how the implementation process.

In conclusion, the present study has confirmed in a real life setting of primary practices that symptoms reported by smoker or ex-smoker patients are not reliable at discriminating COPD from non-COPD. Another challenge that became obvious while implementing the spirometry testing was the impression that any added test was time consuming. Despite being a simple test and the proven role that spirometry has, the lack of allocated time and training of healthcare professionals makes the implementation of this test challenging. For primary care physicians to have access to spirometry, some adjustments will need to be made in our current system. Having trained health professional working in primary care offices and helping with spirometry testing can improve rate of early diagnosis of symptomatic COPD patients in family physician offices.

## Figures and Tables

**Table 1:** Baseline information on recruitment family practices and factors influencing participation.

	Hospital clinics *N* = 1	Community clinics *N* = 4	Private clinics *N* = 7
Health professional per clinic			
Physicians	15	1–11	1–5
Nurses	2	1-2	0–2

Spirometry done by			
Nurse from the clinic	No	Yes	No
Respiratory therapist sent to the clinic	Yes	Yes	Yes

Facilitating factors	Great awareness of the disease and hence already using spirometry	Available nurse to do spirometry	Access to large population of elderly patients
	Direct, frequent contacts with respirologists	Physician has more time per patient	Champion in the team

Limiting factors	Most patients referred with a diagnosis of COPD already	Small volume of patients	Physician has limited visit time per patient (large volume of patients)
	Many different doctors attending the clinic	High immigrant population (language barriers)	Often no personnel available for spirometry
	Nurses available, but overloaded	Younger population	

**Table 2:** Symptoms reported by patients according to the spirometry diagnosis of COPD.

	No COPD *N* = 196	COPD (GOLD I) *N* = 18	COPD (GOLD ≥II) *N* = 32	Overall *P* values
Age (in year), mean (SD)	54.97 (10.34)	58.33 (10.59)	59.88 (8.68)	0.006
Smoking pack years, mean (SD)	31.26 (16.99)	38.06 (10.59)	44.59 (24.99)	0.001
FEV_1_, % predicted, mean (SD)	90.88 (15.14)	88.06 (7.89)	60.92 (13.09)	<0.001
FEV_1_/FVC %, mean (SD)	82.43 (12.98)	64.84 (3.90)	58.56 (8.79)	<0.001
Symptoms, *n* (%)				
Cough	115 (58.67)	11 (61.11)	20 (62.50)	0.908
Cough with sputum	75 (38.27)	8 (44.44)	16 (50.00)	0.424
Dyspnea MRC ≥ 2 scale	127 (64.80)	10 (55.56)	24 (75.00)	0.348
Wheezing	75 (38.27)	8 (44.44)	15 (46.88)	0.599
Respiratory infections*	37 (18.88)	3 (16.67)	9 (28.17)	0.448
Number of symptoms				
0	6 (3.06)	0 (0.0)	0 (0.00)	0.456
1	59 (30.10)	6 (33.33)	6 (18.75)	0.384
2	60 (30.61)	7 (38.89)	12 (37.50)	0.604
3+	71 (36.22)	5 (27.78)	14 (43.75)	0.517

*The infection rate refers to the respiratory infection in the last year.

## References

[1] Murray CJL, Lopez AD (1997). Alternative projections of mortality and disability by cause 1990–2020: Global Burden of Disease Study. *The Lancet*.

[2] Qaseem A, Wilt TJ, Weinberger SE (2011). Disease: a clinical practice guideline update from the American College of Physicians, American College of Chest Physicians, American Thoracic Society, and European Respiratory Society. *Annals of Internal Medicine*.

[3] O’Donnell DE, Aaron S, Bourbeau J (2007). Canadian Thoracic Society recommendations for management of chronic obstructive pulmonary disease—2007 update. *Canadian Respiratory Journal*.

[4] Hill K, Goldstein RS, Guyatt GH (2010). Prevalence and underdiagnosis of chronic obstructive pulmonary disease among patients at risk in primary care. *Canadian Medical Association Journal*.

[5] Walters JA, Haydn Walters E, Nelson M (2011). Factors associated with misdiagnosis of COPD in primary care. *Primary Care Respiratory Journal*.

[6] Bourbeau J, Sebaldt RJ, Day A (2008). Practice patterns in the management of chronic obstructive pulmonary disease in primary practice: The CAGE Study. *Canadian Respiratory Journal*.

[7] Siafakas NM, Vermeire P, Pride NB (1995). Optimal assessment and management of chronic obstructive pulmonary disease (COPD). *European Respiratory Journal*.

[8] Burdon JGW, Pain MCF, Rubinfeld AR, Nana A (1994). Chronic lung disease and the perception of breathlessness: a clinical perspective. *European Respiratory Journal*.

[9] van den Boom G, van Schayck CP, Rutten-van Mölken MPMH (1998). Active detection of chronic obstructive pulmonary disease and asthma in the general population: results and economic consequences of the DIMCA program. *American Journal of Respiratory and Critical Care Medicine*.

[10] Holleman DR, Simel DL (1995). Does the clinical examination predict airflow limitation?. *The Journal of the American Medical Association*.

[11] Melbye H, Aaraas I, Hana J, Hensrud A (1998). Improving pulmonary auscultation as a tool in the diagnosis of bronchial obstruction—results of an educational intervention. *Scandinavian Journal of Primary Health Care*.

[12] Kaplan A (2008). Systems for the management of respiratory disease in primary care—an international series: Canada. *Primary Care Respiratory Journal*.

[13] Glasgow N (2008). Systems for the management of respiratory disease in primary care—an international series: Australia. *Primary Care Respiratory Journal*.

[14] Anthonisen NR, Wooldrage K, Manfreda J (2005). Use of spirometry and respiratory drugs in Manitobans over 35 years of age with obstructive lung diseases. *Canadian Respiratory Journal*.

[15] Radin A, Cote C (2008). Primary care of the patient with chronic obstructive pulmonary disease-part 1: frontline prevention and early diagnosis. *American Journal of Medicine*.

[16] Mahler DA (1998). Dyspnoea in chronic obstructive pulmonary disease. *Monaldi Archives for Chest Disease*.

[17] Rabe KF, Hurd S, Anzueto A (2007). Global strategy for the diagnosis, management, and prevention of chronic obstructive pulmonary disease: GOLD executive summary. *American Journal of Respiratory and Critical Care Medicine*.

[18] Knudson RJ, Lebowitz MD, Holberg CJ, Burrows B (1983). Changes in the normal maximal expiratory flow-volume curve with growth and aging. *American Review of Respiratory Disease*.

[19] Walters JA, Hansen EC, Johns DP, Blizzard EL, Walters EH, Wood-Baker R (2008). A mixed methods study to compare models of spirometry delivery in primary care for patients at risk of COPD. *Thorax*.

[20] Jones RCM, Dickson-Spillmann M, Mather MJC, Marks D, Shackell BS (2008). Accuracy of diagnostic registers and management of chronic obstructive pulmonary disease: the Devon primary care audit. *Respiratory Research*.

[21] Chavez PC, Shokar NK (2009). Diagnosis and management of chronic obstructive pulmonary disease (COPD) in a primary care clinic. *Journal of Chronic Obstructive Pulmonary Disease*.

[22] Lin K, Watkins B, Johnson T, Rodriguez JA, Barton MB (2008). Screening for chronic obstructive pulmonary disease using spirometry: summary of the evidence for the U.S. preventive services task force. *Annals of Internal Medicine*.

[23] Schermer TRJ, Verweij EHA, Cretier R, Pellegrino A, Crockett AJ, Poels PJP (2012). Accuracy and precision of desktop spirometers in general practices. *Respiration*.

[24] Schermer TRJ, Crockett AJ, Poels PJP (2009). Quality of routine spirometry tests in Dutch general practices. *The British Journal of General Practice*.

